# Physiological State and Learning Ability of Students in Normal and Virtual Reality Conditions: Complexity-Based Analysis

**DOI:** 10.2196/17945

**Published:** 2020-06-01

**Authors:** Mohammad H Babini, Vladimir V Kulish, Hamidreza Namazi

**Affiliations:** 1 School of Engineering Monash University Subang Jaya Malaysia; 2 Faculty of Mechanical Engineering Czech Technical University in Prague Prague Czech Republic

**Keywords:** virtual reality, learning ability, brain, facial muscle, fractal theory

## Abstract

**Background:**

Education and learning are the most important goals of all universities. For this purpose, lecturers use various tools to grab the attention of students and improve their learning ability. Virtual reality refers to the subjective sensory experience of being immersed in a computer-mediated world, and has recently been implemented in learning environments.

**Objective:**

The aim of this study was to analyze the effect of a virtual reality condition on students’ learning ability and physiological state.

**Methods:**

Students were shown 6 sets of videos (3 videos in a two-dimensional condition and 3 videos in a three-dimensional condition), and their learning ability was analyzed based on a subsequent questionnaire. In addition, we analyzed the reaction of the brain and facial muscles of the students during both the two-dimensional and three-dimensional viewing conditions and used fractal theory to investigate their attention to the videos.

**Results:**

The learning ability of students was increased in the three-dimensional condition compared to that in the two-dimensional condition. In addition, analysis of physiological signals showed that students paid more attention to the three-dimensional videos.

**Conclusions:**

A virtual reality condition has a greater effect on enhancing the learning ability of students. The analytical approach of this study can be further extended to evaluate other physiological signals of subjects in a virtual reality condition.

## Introduction

Virtual reality refers to the subjective sensory experience of being immersed in a computer-mediated world. Accumulating evidence [1,2] points to the exciting opportunity and potential of integrating virtual reality technology in education environments, which can add elements of reality to improve understanding of complex subjects such as the life sciences (eg, biology and anatomy) compared to traditional classes in which students must imagine the structures for comprehension. In addition, students have been shown to pay more attention to the lecturer when making direct eye contact [3]. However, a lecturer is only able to look at one or two students at a time during a lecture. Therefore, presenting a virtual image of a lecturer to students might increase their attention and consequently improve their learning ability, which can be applied to an electronic learning environment.

Along with the growing empirical evidence that virtual reality is a valuable learning tool, further investigations are needed to study how the use of virtual reality can improve the learning ability of students. In addition, few studies have focused on changes in physiological signals to understand the effect of virtual reality on the human body. Previous studies in this field have compared the brain reaction of anxious participants at rest and under a virtual reality condition [4], compared electroencephalogram (EEG) signals in virtual reality and the traditional display condition [5], analyzed brain activity in response to increasing levels of task complexity in virtual reality [6], employed a deep-learning approach to improve the rate of excitement to well above the 90% accuracy level [7], and analyzed reactions of the heart and brain in different virtual reality environments [8].

The aim of the present study was to investigate the attention and learning ability of students using virtual reality technology. We also investigated the variability of students’ physiological state (facial reaction) under the virtual reality condition. To study the attention and learning ability of students, we recorded their brain signals (ie, EEG signals) and to study facial reactions, we recorded their electromyography (EMG) signals. For comparison, we also recorded the brain signals and facial reactions of the students in a traditional classroom learning condition. EMG was used to capture the engagement of facial muscles during visual perception, which we expected to be more strongly affected under the virtual reality condition.

Since both EEG and EMG signals have complex patterns, we adopted complexity theory for our analysis. In other words, the concept of complexity was employed to define the structure of EEG and EMG signals. Complexity theory can help to characterize the behavior of a system with many parts that interact with each other in highly variable manners [9]. Specifically, we analyzed the recorded EEG and EMG signals using fractal theory, which can be used to quantify the complexity of a system (EEG and EMG signals in this case). Fractals are self-similar or self-affine objects that have complex structures [10]. A self-similar fractal has the same scaling exponent at every scale, whereas a self-affine fractal has different values of the scaling exponent at different scales. EEG and EMG signals are self-affine fractals that have a nonlinear structure. An object with a greater fractal dimension (as a measure of complexity) has a greater level of complexity [11]. Several studies have analyzed different types of physiological signals using fractal theory to date, including analyses of magnetoencephalography [12], galvanic skin response [13], heart rate [14], respiration [15], speech-evoked auditory brainstem response [16], eye movement [17], and human DNA [18]. Similarly, many studies have applied fractal analysis to investigate the nonlinear structure of EEG signals under different conditions, including the influence of auditory [19,20], olfactory [21], and visual [22,23] stimuli; brain diseases [24]; body movements [25,26]; and aging [27].

Some previous studies have also applied fractal theory to analyze EMG signals, including a decoded finger [28,29], hand [29-31], and functional movements and force patterns [29], along with analysis of the effect of complexity of walking on a path with respect to the leg muscle reaction [32]. However, to our knowledge, only one study has employed fractal theory to analyze the facial muscle reaction to date [33].

To analyze and compare the physiological conditions of subjects in virtual reality versus traditional class conditions, we used fractal analysis to relate the complexity of EEG and EMG signals to the nature of the viewed videos.

## Methods

### Study Design

We aimed to analyze students’ physiological state and learning ability under the three-dimensional (3D) virtual reality condition in comparison to those recorded under the traditional two-dimensional (2D) condition. For analysis of the physiological state, we chose EEG and EMG signals as indicators of the brain and muscle response, respectively. EMG signals were selected for the facial muscle reaction since the subjects were stimulated using visual stimuli. For this purpose, we used fractal theory to analyze the complexity of facial EMG and EEG signals. The fractal dimension, as the main quantitative measure of fractal theory, indicates the complexity of the process in which greater values of a fractal dimension reflect greater complexity of the object.

Various methods have been developed to calculate the fractal dimension, which are mainly based on the entropy concept. In this study, we used the box-counting method to calculate the fractal dimension [34]. In the box-counting algorithm, the object of interest is covered with boxes of the same size (ε). The number of boxes (*N*) required to cover the object is then counted. This process is repeated several times, while the box size keeps changing in each step. Finally, the slope of the regression line fitted to a log-log plot of the number of boxes versus the scale is calculated as an estimate of the fractal dimension for the object under consideration [35]:


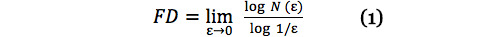


Equation (2) defines the so-called generalized fractal dimension of order *c* [35]:


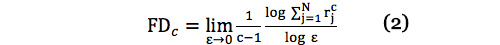


where 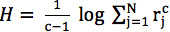 is the Rényi entropy of order *c*, and the probability of occurrence (*r_j_*) is defined as:


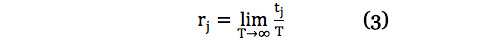


In Equation (3), the total time of the signal value occurrence within the jth value interval is denoted by *t_j_*, whereas *T* represents the total duration of the recorded signal [36].

In this experiment, we showed the students 6 sets of videos (3 videos in the 2D condition and the same 3 videos in the 3D condition), and then investigated the reaction of the brain and facial muscle under both the 2D and 3D conditions using fractal theory to assess the students’ attention to the videos.

In addition, to investigate the learning ability, we designed three questions based on the content of each video (9 questions in total for the 3 videos) that were asked to the students after watching each video in each condition. This questionnaire allowed for assessing the extent to which the students retained and learned the content of the videos.

### Data Collection and Analysis

All procedures from recruiting subjects to conducting the experiment were approved by the Monash University Human Research Ethics Committee (MUHREC; approval number 20965). The study was carried out in accordance with the approved guidelines.

We conducted the experiment with 9 healthy students from Monash University Malaysia. We explained the experiment to the participants and then asked them several questions about their health conditions. Since mental disorders, some medications, as well as drinking beverages that contain alcohol or caffeine affect brain activity and cause inconsistent results, we excluded potential participants within these categories. In addition, participants were excluded if they had consumed beverages containing alcohol or caffeine within 24 hours before the experiments. The students who were deemed to be suitable for experiments signed the consent forms and were included in the study.

We conducted the experiment in a quiet room to isolate the participants from other external stimuli that could potentially affect the recorded EEG and EMG signals. The participants were asked to sit comfortably on a chair during the experiment, and were instructed to focus on watching the videos without engaging in any other task.

**Figure 1:**
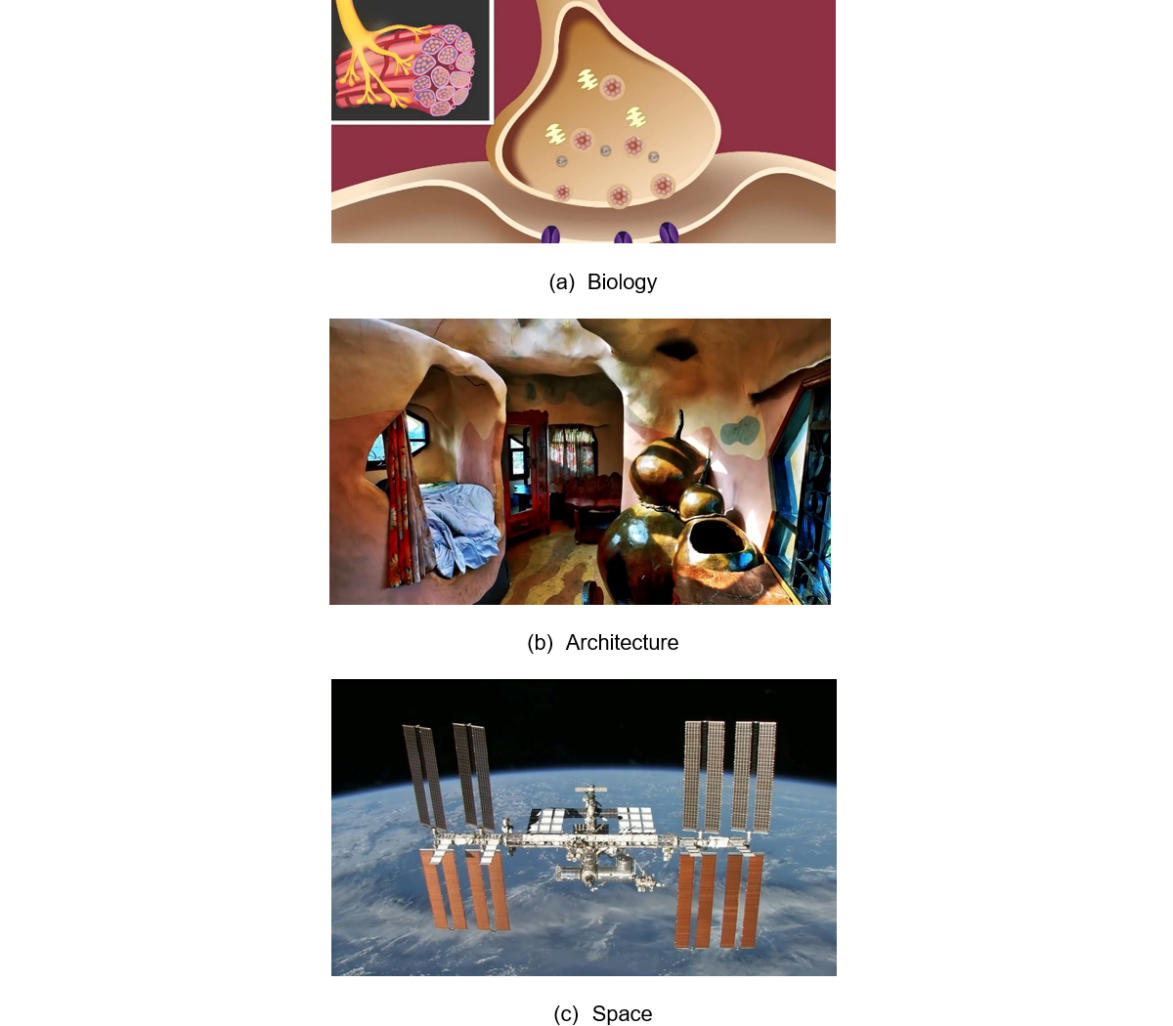
Representative screenshots of scenes from three different videos.

As mentioned above, we chose 6 sets of videos (3 videos in the 2D condition and the same 3 videos in the 3D condition) for our experiment. The 2D videos were selected from YouTube, which were then converted into 3D videos for our experiment. The first and second videos (same content) were about biology (Multimedia Appendix 1 and Multimedia Appendix 2), the third and fourth videos (Multimedia Appendix 3 and Multimedia Appendix 4) were about architecture, and the fifth and sixth videos (Multimedia Appendix 5 and Multimedia Appendix 6) were about space. Some screenshots from these videos are shown in Figure 1.

**Figure 2:**
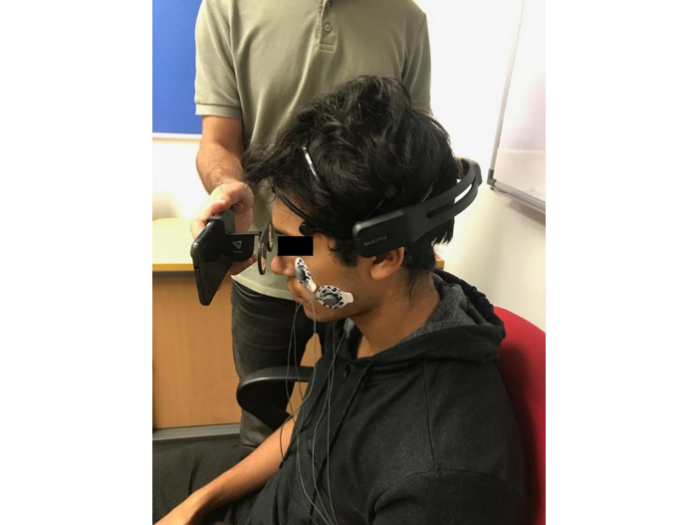
Data collection from a participant.

The videos were displayed to the participants via a mobile phone. The participants watched the 2D videos with the naked eye, whereas the 3D videos were viewed through VeeR MINI VR Glasses (VeeR, Atlanta, GA, USA) (see Figure 2) in front of the mobile phone. We noninvasively recorded EEG and facial EMG signals from the participants using an EMOTIV EPOC+ 14 Channel Mobile EEG headset (Emotiv, San Francisco, CA, USA) and Shimmer EMG device (Shimmer, Ireland) with a sampling frequency of 128 Hz and 256 Hz, respectively. As shown in Figure 2, the EEG device was placed on the participant’s head and five electrodes of the EMG device were connected to the facial muscles.

First, we recorded EEG and EMG signals from the participants for 2 minutes while they watched the first 2D video. When the video was complete, we then asked the participants three questions related to the content. After a 1-minute rest period, the participants watched the first video again in the 3D condition for 2 minutes. The content of this video was identical to that of the 2D video, except that it was presented in 3D mode. Three questions were then asked about the content of the 3D video, followed by another period of rest for 1 minute. We continued this procedure to collect EEG and EMG signals from the participants (along with the responses to content-related questions) with the third, fourth, fifth, and sixth videos (each video lasted for 2 minutes), providing the participants with 1 minute of rest between watching the videos. The data collection was repeated for each participant in the second session to validate the repeatability of results.

Initially, we preprocessed the raw data to remove noise. For this purpose, we wrote a set of codes in MATLAB (MathWorks, Natick, MA, USA) based on the Butterworth filter. The frequency bands of 1-40 Hz and 25-125 Hz were chosen for filtering the EEG and EMG signals, respectively. Of note, two electrodes of the EEG device had some disconnection problems during data collection; therefore, we processed the collected data only from the other 12 electrodes.

After initial filtering, we proceeded with the analysis by computing the fractal dimension of the recorded EEG and EMG signals. The computation of the fractal dimension was based on the box-counting algorithm using boxes with sizes (1/2, 1/4, 1/8, etc) as the scaling factor. Although we recorded 120 seconds of data during each period of watching the 2D and 3D videos, we analyzed only 118.2 seconds of each dataset. This selection was due to the fact that the devices did not always have a consistent sampling frequency, which caused the data recording to be less than 2 minutes long, leading to a difference of a few seconds in the duration of collected data among some participants.

After confirming the normal distribution of the data, statistical analysis of the computed fractal dimension for EEG and EMG signals was performed to assess the effect of stimulation on variations of the fractal dimension of EEG and EMG signals using one-way repeated-measures analysis of variance (ANOVA). We also conducted the Student *t* test to compare the difference in mean values of EEG or EMG signals between the 2D and 3D condition. *P*<.05 was considered to reflect a statistically significant difference in our analysis.

## Results

The variations of fractal dimensions of EEG signals for the first to the sixth visual stimuli are shown in Table 1. As mentioned above, the first, third, and fifth stimuli refer to the 2D condition, whereas the second, fourth, and sixth stimuli refer to the 3D condition.

**Table 1:** Fractal dimension of EEGa signals with the first to sixth stimuli.

Stimulus	Fractal dimension of EEG signal
First	1.7027
Second	1.7266
Third	1.7196
Fourth	1.7222
Fifth	1.6928
Sixth	1.7272

^a^EEG: electroencephalogram.

Based on the result of ANOVA (*F*=7.6334, *P*<.001), the effect of stimulation (2D and 3D) on variations of fractal dimensions of the EEG signal was significant. As shown in Table 1, for all stimuli, the EEG signal recorded from the participants in the 3D condition had a greater fractal dimension compared to that recorded in the 2D condition. Since the fractal dimension reflects the complexity of the signal, this result indicated that the EEG signal is more complex in response to 3D visual stimuli compared to 2D visual stimuli. In other words, the human brain becomes more engaged with a stimulus when it is presented in the 3D condition compared to the 2D condition. Differences between the mean values of the EEG signal from the first and second stimuli (*P*=.001) and from the fifth and sixth stimuli (*P*<.001) were greater than the difference between the third and fourth stimuli (*P*=.72). This suggested that the participants’ brains were more engaged with the second and sixth stimuli compared to the third stimuli. This result is reasonable given that the second and sixth stimuli mainly contained animated scenes, whereas the fourth stimulus included more photos with less animated scenes. Therefore, the difference between the fractal dimension of the EEG signals in the third and fourth stimuli was lower than that observed under the other conditions.

The variations of the fractal dimension of the EMG signals for the first to the sixth visual stimuli are summarized in Table 2.

**Table 2:** Fractal dimension of EMGa signals with the first to sixth stimuli.

Stimulus	Fractal dimension of EMG signal
First	1.2361
Second	1.2594
Third	1.2554
Fourth	1.2580
Fifth	1.2488
Sixth	1.2675

^a^EMG: electromyography.

Based on the result of ANOVA (*F*=0.2468, *P*=.94), the effect of stimulation (2D and 3D) on variations of the fractal dimension of the EMG signals was not significant. Upon receiving a visual stimulus (2D or 3D), the brain processes the stimulus and then sends the impulses to the facial muscles. Therefore, the stimulus should have a greater effect on the brain than on the facial muscles, which explains why there was a significant effect of the stimuli on variations of EEG signals but not on the facial muscles.

As shown in Table 2, for all stimuli, the EMG signal had a greater value of the fractal dimension in response to 3D videos compared to 2D videos, indicating that the EMG signal is more complex in response to 3D videos compared to 2D videos. In other words, the facial muscles are more engaged with stimuli where they are presented in 3D rather than in 2D. In addition, the difference between the mean values of the EMG signal in the first and second stimuli (*P*=.43) and the fifth and sixth stimuli (*P*=.56) was greater than that between the third and fourth stimuli (*P*=.93). This indicates that the participants’ facial muscles were more engaged with the second and sixth stimuli compared to the third stimuli. As mentioned above, this difference can be explained by the content of the videos, in which the second and sixth stimuli contained more animated scenes compared to the fourth video.

Despite these differences among videos, there was no significant difference in the fractal dimension of the EMG signal between each pair of stimuli. This suggests that although presenting the videos in 3D caused some changes in the muscle reaction, these changes were not substantial. Comparison of the results for EMG and EEG signals indicates that changing the visual stimulus from 2D to 3D could cause significant variations in the complexity of the EEG signal, but not in the EMG signal. Therefore, changes in the state of the brain are greater when changing a visual stimulus from 2D to 3D.

Moreover, evaluating the relationship between variations of EEG and EMG signals can provide further insight. The brain controls all parts of the human body, including the reactions of the facial muscle. When exposed to 2D or 3D videos as visual stimuli, the brain sends impulses to the facial muscles. Therefore, when the brain is more engaged with the stimuli, the muscle reaction will also be greater, which is reflected in the greater variations in the fractal dimension of the EMG signal.

The rate of correct responses to the questions posed after watching the 3D video was 92.60%, which was higher than that obtained after the 2D videos at 80.87%. This difference suggested that the 3D videos resulted in greater attention paid to the details of videos and therefore increased the learning ability of the students.

## Discussion

In this study, we compared the effect of virtual reality on students’ learning ability and physiological state with those recorded in a normal 2D condition based on watching 3 sets of videos each presented in 2D and 3D. We simultaneously recorded EEG and facial EMG signals of the participants during stimulation. Overall, the EEG and EMG signals had greater fractal dimensions in the 3D video condition, indicating that both the brain and facial muscles have a greater reaction to 3D videos compared to 2D videos. In addition, videos with more animated scenes resulted in a significantly greater brain reaction compared with that resulting from watching a video with less animated scenes, as reflected by the lack of a significant difference in the fractal dimension of EEG signals between 2D and 3D conditions. For the EMG analysis, although the 3D condition caused greater reaction in the facial muscle, there was no significant difference from the reaction recorded under the 2D condition.

We also examined the learning ability of the students after watching each video by asking them several content-related questions, demonstrating improved learning ability after watching 3D videos than 2D videos (92.60% vs 80.87% correct answers). These results clearly showed that students pay more attention to videos when they are presented in 3D. The present study offers a step forward compared to previous studies that only analyzed the learning ability or brain reaction [4-7] in a virtual reality condition without considering the reaction of facial muscles and investigating how that reaction correlates with brain activity.

The method of analysis employed in this study can be extended to investigate other physiological signals of students in a virtual reality condition. For instance, we can analyze how the heart rate changes in a 3D condition compared to a 2D condition. We can also expand this work by applying other types of stimuli, including olfactory stimuli, while students are watching videos in the virtual reality condition and investigate the effect of these additional stimuli on their learning ability. Developing a model between input (videos) and outputs (human physiological signals) is another important aspect of future work in this regard. For this purpose, we can benefit from different tools such as machine learning [37-39] and fractional-based mathematical equations [40]. Such analysis could allow for predicting human conditions (physiological signals) before exposure to different stimuli, providing guidance on the types of videos and characteristics of videos that are most likely to arouse the attention of students and facilitate learning. These efforts therefore have great importance in advancing research on students’ learning ability and can provide strong recommendations to educational institutions.

## Appendix

Multimedia Appendix 1 2D-biology.

Multimedia Appendix 2 3D-biology.

Multimedia Appendix 3 2D Architecture.

Multimedia Appendix 4 3D Architecture.

Multimedia Appendix 5 2D Space.

Multimedia Appendix 6 3D Space.

## Abbreviations

2D: two-dimensional
3D: three-dimensional
ANOVA: analysis of variance
EEG: electroencephalogram
EMG: electromyography
